# Low glucose microenvironment of normal kidney cells stabilizes a subset of messengers involved in angiogenesis

**DOI:** 10.14814/phy2.12253

**Published:** 2015-01-19

**Authors:** Elodie de Laplanche, Asma Boudria, Estelle Dacheux, Anne Vincent, Nicolas Gadot, Fouzia Assade, Katy Le Corf, Xavier Leroy, Florence Mège Lechevallier, Béatrice Eymin, Nicole Dalla Venezia, Hélène Simonnet

**Affiliations:** Université de Lyon, Lyon, F‐69000, France; Université Lyon 1, Lyon, F‐69000, France; Inserm U1052, Centre de Recherche en Cancérologie de Lyon, Lyon, F‐69000, France; CNRS UMR5286, Centre de Recherche en Cancérologie de Lyon, Lyon, F‐69000, France; Institut Albert Bonniot Equipe 2 Bases Moléculaires de la Progression des Cancers du Poumon, INSERM U823/Université Joseph Fourier, Grenoble, F‐38000, France; Department of Pathology, Hôpital Edouard Herriot, Lyon, F‐69000, France; Institut de Pathologie, CHRU, Faculté de Médecine, Université de Lille, Lille, F‐59000, France

**Keywords:** Glucose sensing, HIF1A, metabolism, normal renal tubule, VEGFA

## Abstract

As glucose is a mandatory nutrient for cell proliferation and renewal, it is suspected that glucose microenvironment is sensed by all cell types to regulate angiogenesis. Several glucose‐sensing components have been partially described to respond to high glucose levels. However, little is known about the response to low glucose. Here, we used well‐differentiated isolated normal rat renal tubules under normal oxygenation conditions to assess the angiogenic response to low glucose. In apparent paradox, but confirming observations made separately in other models, high glucose but also low glucose increased mRNA level of vascular endothelial growth factor A (VEGFA). A subset of mRNAs including hypoxia‐inducible factor 1A (HIF1A), angiopoietin receptor (TIE‐2), and VEGF receptor 2 (FLK1) were similarly glucose‐sensitive and responded to low glucose by increased stability independently of HIF1A and HIF2A proteins. These results contribute to gain some insights as to how normal cells response to low glucose may play a role in the tumor microenvironment.

## Abstract (precis)

As glucose is a mandatory nutrient for cell proliferation and renewal, it is suspected that glucose microenvironment is sensed by all cell types to regulate angiogenesis. If several glucose‐sensing components have been partially described to respond to high glucose levels, little is known about the response to low glucose. Here, we used well‐differentiated isolated normal rat renal tubules under normal oxygenation conditions to assess the angiogenic response to low glucose.

## Introduction

Glucose is a mandatory nutrient for cell proliferation and renewal (Locasale and Cantley 2011). It has been suspected that glucose microenvironment is sensed by all cell types in order to regulate the pathways involved in these functions. While using little or no oxygen, many cancer cells take up glucose with avidity and depend on this nutrient for their growth (Rodriguez‐Enriquez and Moreno‐Sanchez 1998; Ortega et al. 2009). Therefore, it has long been pointed that neoangiogenesis serves the purpose of providing glucose rather than oxygen in tumors. Several glucose‐sensing components have been partially described in different cell types, such as O‐glycosylation of regulatory proteins (Butkinaree et al. 2010), glucose sensing at the membrane level by GLUT2 (Stolarczyk et al. 2007), the AMPK/mTOR‐dependent protein synthesis (Lee et al. 2012), and the CHREBP transcription factor in liver (Havula and Hietakangas 2012). All these components have been mainly shown to respond to high glucose levels. In contrast, we still do not know whether and how low glucose concentrations induce a response in order to restore nutrient supply. Yet, as early as 1995, the group of Keshet had shown that the vascular endothelial growth factorA (VEGFA) is upregulated by low glucose (increased by shortage) in glial cell spheroids considered as stem cells, both at the mRNA and protein levels, through mRNA stabilization (Shweiki et al. 1995; Stein et al. 1995). In apparent contrast, in other organs such as kidney tubule and retina, it is high glucose (increased by abundance) that has been mentioned to increase VEGFA transcript and protein levels (Braun et al. 2001; Kane et al. 2005; Kim et al. 2005), and, in kidney tubule, hyperglycemia favors hypertrophy (Zhang et al. 2007). To date, the response of other angiogenic factors to low glucose remains unclear.

The main known regulator of angiogenesis and energy metabolism in normal cells is the hypoxia‐inducible factor (HIF) (Semenza 2012). HIF binding to DNA induces the transcription of at least 100 mRNAs involved in neoangiogenesis (e.g., VEGFA, VEGF receptor 2 (FLK1), angiopoietin receptor (TIE‐2)), in glycolysis (e.g., glucose facilitated transporter 1 (GLUT1), Aldolase A), in extracellular matrix remodeling and in survival (Semenza 2002). VEGFA and its main receptor FLK1 are involved in several other cellular functions including vascular permeability, cell division and survival, endothelium budding, but also endothelial cell migration (Koch and Claesson‐Welsh 2012). Moreover, it is now recognized that VEGF receptors, believed so far to be restricted to endothelial cells, are expressed widely (Koch and Claesson‐Welsh 2012). Their regulator HIF contains two subunits, HIFA and HIFB, the former being sensitive to oxygen pressure that controls both its protein stability and transcriptional activity. Under normal oxygen content, HIFA is hydroxylated (Maxwell and Ratcliffe 2002) and recognized by the tumor suppressor protein product of von Hippel‐Lindau gene (VHL) (Li and Kim 2011), causing HIFA ubiquitination and subsequent degradation by the proteasome (Kaelin 2005). Under low oxygen or when VHL is defective such as in clear cell renal carcinoma, HIFA protein is stable (Maxwell et al. 1999) and forms with its partner HIFB a dimer that binds DNA on the Hypoxia Response Elements (HREs). Three isoforms of the regulated HIFA subunit have been described, each being encoded by a separate gene. HIF1A and HIF2A mRNAs are expressed in all organs tested, including the kidney (Heidbreder et al. 2003). The HIF2A protein presents good structural and functional homologies with HIF1A and is also downregulated by VHL, yet its roles and targets are different in some aspects (Keith et al. 2011). Regulation of HIF3A is less documented. This protein has also been found in kidney, and several splicing variants have been described (Heidbreder et al. 2003; Heikkila et al. 2011). HIF3A protein expression is generally considered as an inhibitor of the function of the other family members (Hara et al. 2001; Maynard et al. 2005). Finally, the less well‐known field of HIFA biology is the regulation of its messengers. Studies in tumor cells showed that HIF1A and HIF2A mRNA levels are not regulated by oxygen (Maxwell et al. 1999; Heidbreder et al. 2003). However, it is not known whether they are sensitive to other nutrients.

In this study, we decided to further investigate the metabolic adaptation of normal nontransformed kidney cells to glucose microenvironment. We had previously shown that normal tubules suspensions shift from lactate utilization to lactate production when not efficiently shaken in a gas phase of 20% oxygen (Bolon et al. 1997) (de Laplanche et al. 2006). Using this model, we had previously described transcriptome modifications 4 h after oxygenation changes. Hence, we had shown that *hypoxia*, which in this model is far from the classical but drastic oxygen deprivation achieved with unstirred cells cultured below 1% oxygen in the gas phase, induces many marked changes in tubule specific mRNAs (de Laplanche et al. 2006). In the present work, we took advantage of this model of isolated renal cortical tubules to investigate the response to glucose shortage. The results showed that low glucose in the microenvironment of normal kidney cells increases a series of mRNAs involved in angiogenesis including VEGFA, HIF1A, and HIF2A themselves. VEGFA protein was upregulated and its mRNA was stabilized under low glucose in the presence of oxygen independently of HIF1A protein.

## Material and Methods

### Ethics statement

The study was carried out in strict accordance with the recommendations of the French National Ethics Committee. The protocol was approved by the Comité d'Ethique en Experimentation Animale (CE2A) of the University Claude Bernard Lyon 1 (agreements UCBL1 no BH2007‐07 and no DR2013**‐**02).

### Preparation and incubation of isolated rat renal tubule suspensions

Euthanasia procedure was as follows: animals received a lethal injection of pentobarbital before kidneys were removed. Renal cortical tubule suspensions were isolated by collagenase digestion from pooled kidney cortices of four male Sprague–Dawley rats (Charles River, Les Oncins, France), as previously described (de Laplanche et al. 2006). They were incubated in DMEM/F12 medium containing 25 mmol/L bicarbonate, 10 mmol/L HEPES at pH 7.4, 5 mmol/L glucose (control condition), 0.5 mmol/L lactate, 4 mmol/L glutamine, and 20 mg/L egg‐white lysozyme as an antibacterial agent, in stoppered flasks equilibrated with 95% air/5% CO_2_ and shaken at 37°C under variable protein concentrations and variable incubation volumes as described previously (de Laplanche et al. 2006) to achieve reproducible normoxia or hypoxia. At the end of the incubation time, pO_2_, pCO_2_, and pH of the liquid phase were measured with a gas analyzer (Radiometer ABL5) and the remaining suspension was centrifuged. After 4 h, mean final value of pO_2_ was 42 ± 4 mm Hg in the medium, pCO_2_ 45 ± 5 mmHg, pH 7.37 ± 0.02, and glucose 5.0 ± 1.3 (data from four experiments). All final pO_2_ values in liquid phase are reported in Fig. 4A. The pelleted tubules were stored at −80°C for RNA extraction or for protein analysis. The supernatants aliquots were immediately stored at −80°C for protein determination or deproteinized and stored at −20°C for further glucose and lactate determinations.

### RNA analyses

Transcripts relative abundances were determined as previously described (de Laplanche et al. 2006). Briefly, total RNA was extracted with Trizol reagent (Life Technologies) and double‐stranded cDNAs synthesized. Real‐time or semiquantitative RT‐PCR was performed and the values normalized to that of the ribosomal protein encoding L32, which is not a HIF target. Primer sequences and elongation temperatures were as follows: HIF1A, (region which is not complementary to aHIF), FOR (A1): GGACAAGTCACCACAGGACA, REV (A2): GGAGAAAATCAAGTCGTGCTG, 65°C, HIF2A, FOR: GCGACAATGACAGCTGACAAGG, REV: GTCCCATGAACTTGCTGATGTTT, 66°C, VEGF A, 188 variant, FOR: CCCGGTTTAAATCCTGGTGGAGCG, REV: CGCCTTGGCTTGTCACATCTGC, 66°C, TIE‐2, FOR: ATGGACTCTTTAGCCGGCTTA, REV: CCTTATAGCCTGTCCTCGAA, 56°C, FLK1, FOR: GCAAGAGCAGAGACACTCTTC, REV: CTGGCATCATAAGGCAAGCG, 56°C, GLUT1, FOR: CAGGCTCCATTTAGGATTCGCC, REV: CTCAGCCTCCGAGGTCCTTCTC, 64°C, Aldolase A, FOR: CAGGAAGCATTGCCAAGCGCC, REV: GACACTGTACCAGAAGGC, 68°C. *Controls*. L32, FOR: GTGAAGCCCAAGATCGTCAA, REV: TTGTTGCACATCAGCAGCAC, 60°C, 18S, FOR: TGAACCCCATTCGTGAT, REV TACAAAGGGCAGGGACTTA, 53°C. Where semiquantitative RT‐PCR was performed, representative agarose gels were shown and the intensity of bands corresponding to PCR products was quantified using ImageQuant software^TM^ (Molecular Dynamics, Sunnyvale, CA).

### Measurement of mRNA half‐lives

Isolated tubule suspensions were preincubated for 2 h under different glucose concentrations to reach a steady state in mRNA metabolism. Then 50 *μ*mol/L of the polymerase II inhibitor 5,6‐Dichlorobenzimidazole 1‐beta‐D‐ribofuranoside (DRB) were added through the stopper to achieve the maximal transcription inhibition as determined in preliminary experiments. Tubules were pelleted for RNA measurement as above. mRNA contents were normalized to that of 18S ribosomal RNA.

### Western blot analyses

After whole tubule pellet lysis with 450 mmol/L NaCl, 50 mmol/L Tris‐EDTA pH 8, 10% glycerol, 0.5% NP40 and inhibitors of proteases and phosphatases, VEGF was analyzed by western blotting using the pan‐VEGF antibody from Santa Cruz Biotechnology (Heidelberg, Germany) (sc‐152) and the anti‐VEGFxxxb antibody from ABCAM (Cambridge, UK) (ab14994). FLK1, TIE‐2, GLUT1, Aldolase A and Tubulin were analyzed by western blotting with antibodies from, respectively, Cell Signaling (2479), Santa Cruz (sc‐9026), ABCAM (ab32551), Cell Signaling (Danvers, MA) (3188), and Calbiochem (Millipore SAS, Molsheim, France) (CP06). HIF1A, HIF2A, and actin (epitope present in the six isoforms of vertebrate actin) were determined in nuclear fraction after tubule lysis (Zou et al. 2001) by western blotting with antibodies from, respectively, Santa Cruz Biotechnology Inc (sc‐10790), ABCAM (ab8365) and Chemicon (Millipore SAS) (MAB1501) as previously described (Hervouet et al. 2005). Protein levels from the blots were evaluated using the gel analysis ImageLab software (BioRad, Hercules, CA) and the ratio of protein levels in the normal glucose supply versus the low and high glucose supplies was represented as fold change.

### Statistical analysis

Results are expressed as mean ± the standard error of mean (SEM). Statistical significance was determined using two‐tailed paired Student's *t* test and *P* values of <0.05 were considered significant.

## Results

### Low glucose increases VEGFA mRNA and a subset of mRNAs involved in angiogenesis and glucose consumption

**Figure 1.**
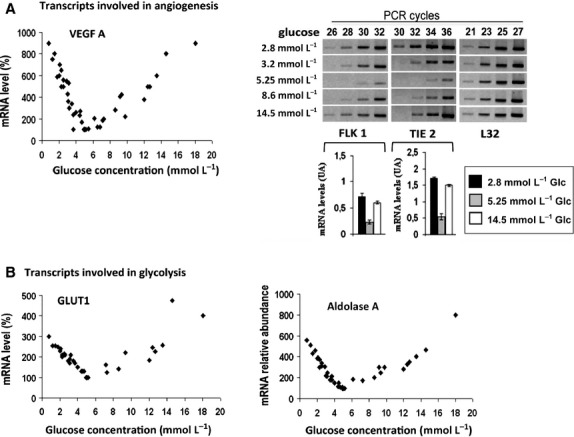
A subset of mRNAs including VEGFA is overexpressed under low and high glucose. Tubule suspensions were incubated for 4 h under a pO_2_ of approximatively 50 mm Hg and various glucose concentrations in four separate experiments. Abundance of VEGFA, GLUT1, and Aldolase A mRNAs was determined by RT‐real‐time PCR. The results were standardized with hypoxia‐insensitive L32 mRNA and expressed as the percentage (%) of the value at normal (5 mmol/L) glucose. Abundance of FLK1 and TIE‐2 mRNAs was determined by semiquantitative RT‐PCR and analyzed on agarose gels (representative experiment). The intensity of the bands corresponding to PCR products obtained under low (2.8 mmol/L), normal (5.25 mmol/L) and high (14.5 mmol/L) glucose were quantified using Quantsoftware^TM^. The values correspond to intercept cycle and represent the mean ± SEM of four independent experiments.

Freshly isolated rat kidney tubules were incubated in the presence of different glucose concentrations and under a mean final pO_2_ value of approximately 40 mm Hg, which is normal for this highly oxidative tissue. Modifying glucose levels did not alter pO_2_ (not shown). We first investigated the effect of glucose on various mRNAs involved in angiogenesis such as those of VEGFA, its main receptor FLK1 and the angiopoietin receptor TIE‐2, as well as on others mRNAs involved in glucose uptake (GLUT1) or in glycolysis (Aldolase A). As expected, compared to normal glucose conditions, high glucose concentration increased VEGFA mRNA level, confirming previous observations in rat kidney in vivo (Braun et al. 2001; Kim et al. 2005). Furthermore, in our model, VEGFA, FLK1, TIE2, GLUT1, and Aldolase A mRNA levels also increased under low glucose (Fig. 1A and B). This effect was specific of these transcripts as L32 mRNA was not sensitive to glucose microenvironment. Therefore, not only high glucose levels but also low glucose levels are able to increase mRNAs implicated in angiogenesis and glucose consumption. These data obtained in normal rat kidney tubules for VEGFA are in agreement with the findings of Stein and coworkers in spheroids of glial cells (Stein et al. 1995).

### Low glucose also increases VEGFA and GLUT1 protein levels

**Figure 2.**
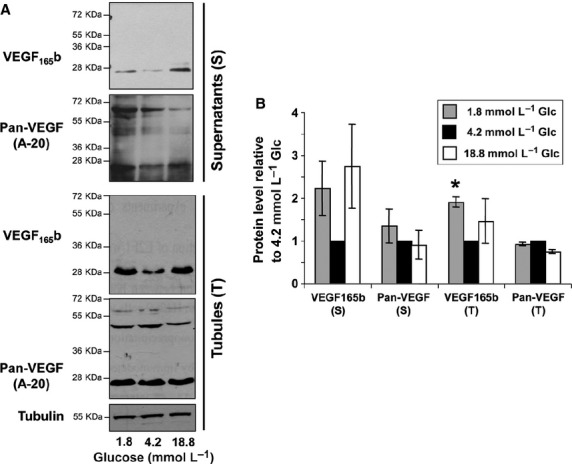
VEGFA is increased under low glucose. (**A) **VEGFA protein levels were determined either with an anti‐pan‐VEGF or an anti‐VEGF165b antibody in lysates of the tubules pellets and in supernatants of the same suspension after 7 h of incubation. Tubulin served as loading control in tubule lysates. No loading control is given for supernatants as no secreted protein can normalize the cell amount. Here equal volumes of supernatants were loaded, issuing from equal amounts of the same tubule suspension. The experiments were repeated two times, and representative results are shown. (B) The intensity of bands corresponding to VEGF (i.e., bands around 30 kDa) was quantified and the amount of VEGF was normalized to the 4.2 mmol/L Glc culture condition (set at 1). The values are mean ± SEM of two independent experiments. *P < 0.05 compared with 4.2 mmol/L Glc. S: Supernatants; T: Tubules.

VEGFA gene is transcribed into several splice variants. The alternative variants containing the 8b exon are termed xxxb (xxx for the size of the protein in number of aminoacids) (Woolard et al. 2009). The xxxb proteins have been shown to display poor angiogenic capacities but are highly expressed in normal kidney tissue (Woolard et al. 2009; Grepin et al. 2012). We therefore analyzed VEGFA protein expression in both intracellular extracts and supernatants of renal tubules, using either an antibody directed against all isoforms (pan‐VEGF) or against the antiangiogenic isoform (VEGF_165_b) (Fig. 2A**)**. The data clearly showed that VEGF_165b_ protein level increased under both low and high glucose conditions in supernatants as well as in tubules. Notably, under low glucose, we observed a neat and statistically significant twofold increase in the level of VEGF_165b_ in intracellular extracts of tubules, as compared to normal glucose conditions (Fig. 2B). Regarding total VEGFA, our experiments showed a 30% increase in the VEGFA levels in supernatants under low glucose but not in tubule extracts. Discrepancy between intracellular and secreted levels of VEGFA had already been reported. For instance, in a very large‐scale study of breast, lung, and colon cancer, it has been very recently shown that plasmatic levels of VEGFA did not correlate with intratumoral levels of VEGFA (Hegde et al. 2013). Therefore, these and our data highly suggest that the intra‐ and extracellular levels of VEGFA are not always correlated. In this study, low and high glucose concentrations might therefore favor VEGFA secretion.

**Figure 3.**
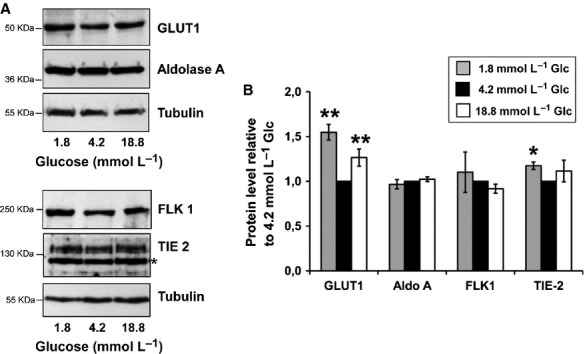
GLUT1 and TIE‐2 are increased under low glucose. (A) GLUT 1, Aldolase A, FLK 1 and TIE‐2 protein levels were determined in lysates of the tubules pellets of the same suspension after 7 h of incubation. Tubulin served as loading control. The asterisk indicates a nonspecific band. The experiments were repeated three times, and representative results are shown. (B) The intensity of bands corresponding to GLUT 1, Aldolase A, FLK 1 and TIE‐2 was quantified and the amount of each protein was normalized to the 4.2 mmol/L Glc culture condition (set at 1). The values are mean ± SEM of three independent experiments. *P < 0.05 and **P < 0.01 compared with 4.2 mmol/L Glc.

The GLUT1 protein was clearly sensitive to high and low glucose, as shown by the increased GLUT1 levels under both glucose conditions (Fig. 3A upper panels). Notably, under low glucose, our experiments showed a neat and statistically significant 50% increase in the GLUT1 levels. In addition, a significant 30% increase was obtained under high glucose, as compared to normal glucose conditions (Fig. 3B). Regarding Aldolase A protein, no change could be detected (Fig. 3A upper panels, and B). This may be explained by the very long half‐life of Aldolase A protein (one to several days (Hopgood et al. 1988)). Concerning TIE‐2 protein, its level was increased, still slightly, under both high and low glucose (Fig. 3A lower panels, and B). This slight, and therefore hard to be statistically significant under western blotting measurement, may account for the TIE‐2 half‐life of 7 h (Bogdanovic et al. 2006). Concerning FLK1 protein, its level was slightly increased under low glucose. It is to note that clear variations in FLK1 levels were expected to be detected since its protein half‐life is 1 h or less (Murakami et al. 2011). However, our data are consistent with the observation previously made by Adham and Coomber who had shown in endothelial cells that glucose lowering to 2 mmol/L does not alter FLK1 protein level or even decreases it, whereas in the meantime its transcript level is increased (Adham and Coomber 2009).

As a whole, our results show that the levels of VEGFA and GLUT1 proteins and to a lesser extent TIE‐2 protein, are regulated in the same way than their encoding mRNAs in response to low glucose in our cellular model.

### HIF1A and HIF2A mRNA levels are not correlated with oxygen concentration, but respond to glucose in parallel with those of angiogenic mRNAs

**Figure 4.**
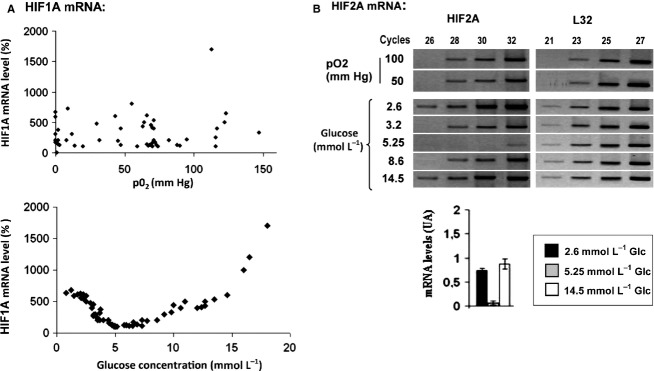
HIF1A and HIF2A mRNA levels are increased under low glucose. Tubule suspensions were incubated as described for 4 h under different conditions of oxygenation and of glucose concentration in four independent experiments. Final pO_2_ and final glucose concentration were determined for each experimental point. mRNA levels were determined by RT‐real time PCR (HIF1A and L32) or by semiquantitative PCR (HIF2A and L32). (A) HIF1Α mRNA values were standardized to L32 mRNA level then expressed in % of their value in control conditions (normoxia and normal glucose). (B) Effect of oxygen (under normal glucose) and of glucose (under a pO_2_ of 50 mm Hg) on HIF2A mRNA levels. Abundance of HIF2A mRNA was determined by semiquantitative RT‐PCR and analyzed on agarose gels (representative experiment).The intensity of the bands corresponding to PCR products obtained under low (2.6 mmol/L), normal (5.25 mmol/L) and high (14.5 mmol/L) glucose were quantified using Quantsoftware^TM^. The values correspond to intercept cycle and represent the mean ± SEM of four independent experiments.

We then searched for mRNA variations of the main regulator of angiogenesis, HIF, focusing on the regulated subunits HIF1A and HIF2A. Experiments were performed under different conditions of oxygenation and different glucose concentrations. Final pO_2_ in liquid phase was determined in parallel with final glucose content in each flask and final values were reported in Fig. 4A (upper panel). The results showed that HIF1A or HIF2A mRNA levels did not correlate with pO2 values, in spite of large variations (Fig. 4A and B, upper panels). This is in agreement with previous studies in renal cancer cell lines and in normal kidney tissue (Maxwell et al. 1999; Heidbreder et al. 2003). In contrast, in response to glucose, HIF1A and HIF2A mRNA levels varied in the same way than the angiogenic and glycolytic mRNAs depicted in Fig. 1 (Fig. 4A and B, lower panels). We noticed that the lowest concentration of glucose yields irregular results, with a fall in HIF1A mRNA level when the final concentration falls below a limit estimated at 0.5 mmol/L glucose (not shown). This fall of HIF1A expression in absence of glucose has already been reported (Vordermark et al. 2005). Above this threshold, there was a clear cut covariation between HIF1A transcript and glucose final content (Fig. 4A, lower panel). Glucose content as low as 3 mmol/L markedly increased intracellular HIF1A transcript level when compared with the reduced level at 5 mmol/L glucose. High glucose concentrations of 7–8 mmol/L, and concentrations up to values frequently reached in diabetes, again increased HIF1A transcript level. The response of HIF2A mRNA paralleled that of HIF1A (Fig. 4B lower panel). Taken together, these results clearly demonstrate that HIFA transcript is tightly regulated by one of the main nutrients, glucose, and notably increased by its shortage.

### VEGFA mRNA changes under low and high glucose are likely HIF‐independent

**Figure 5.**
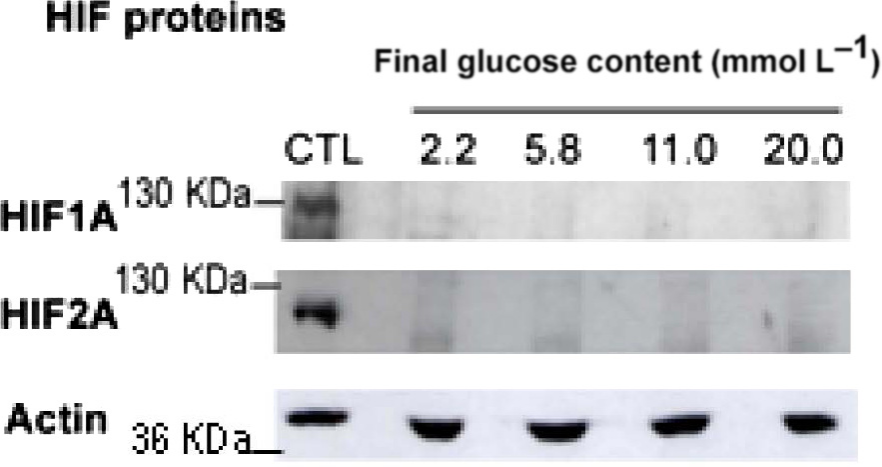
HIF1A and HIF2A proteins are not detectable in normal kidney tubules. Tubules were incubated as in Figs 1 and 4. HIF1A and HIF2A proteins were determined after 4 h by immunoblotting in two separate experiments. Positive control (CTL): proteins extracted from rat hippocampus under hypoxia. Actin served as loading control. A representative experiment is shown.

VEGFA, FLK1, TIE‐2, GLUT1, and Aldolase 1 genes are targeted by the HIF1A and HIF2A transcription factors. Therefore, we asked whether glucose‐induced increase in VEGFA, FLK1, TIE‐2, GLUT1, and Aldolase 1 mRNA levels was correlated with an increase in HIF1A and/or HIF2A protein levels. We were not able to detect a significant amount of HIF1A or HIF2A protein by western blotting in our model of normal cells, whatever glucose concentrations (Fig. 5). Several authors have already reported that HIFA proteins are inconsistently or hardly detectable in normal kidney cortex in vivo (Stroka et al. 2001; Zou et al. 2001; Heidbreder et al. 2003; Rosenberger et al. 2005). This is probably due to oxygen‐sensitive degradation, as HIF1A is detected in the less vascularized medulla zone of the kidney (Zou et al. 2001). Therefore, in this model, glucose‐mediated VEGFA mRNA changes are likely HIF‐independent.

### Low glucose increases half‐lives of HIF1A, VEGFA, and other HIF target mRNAs

**Figure 6.**
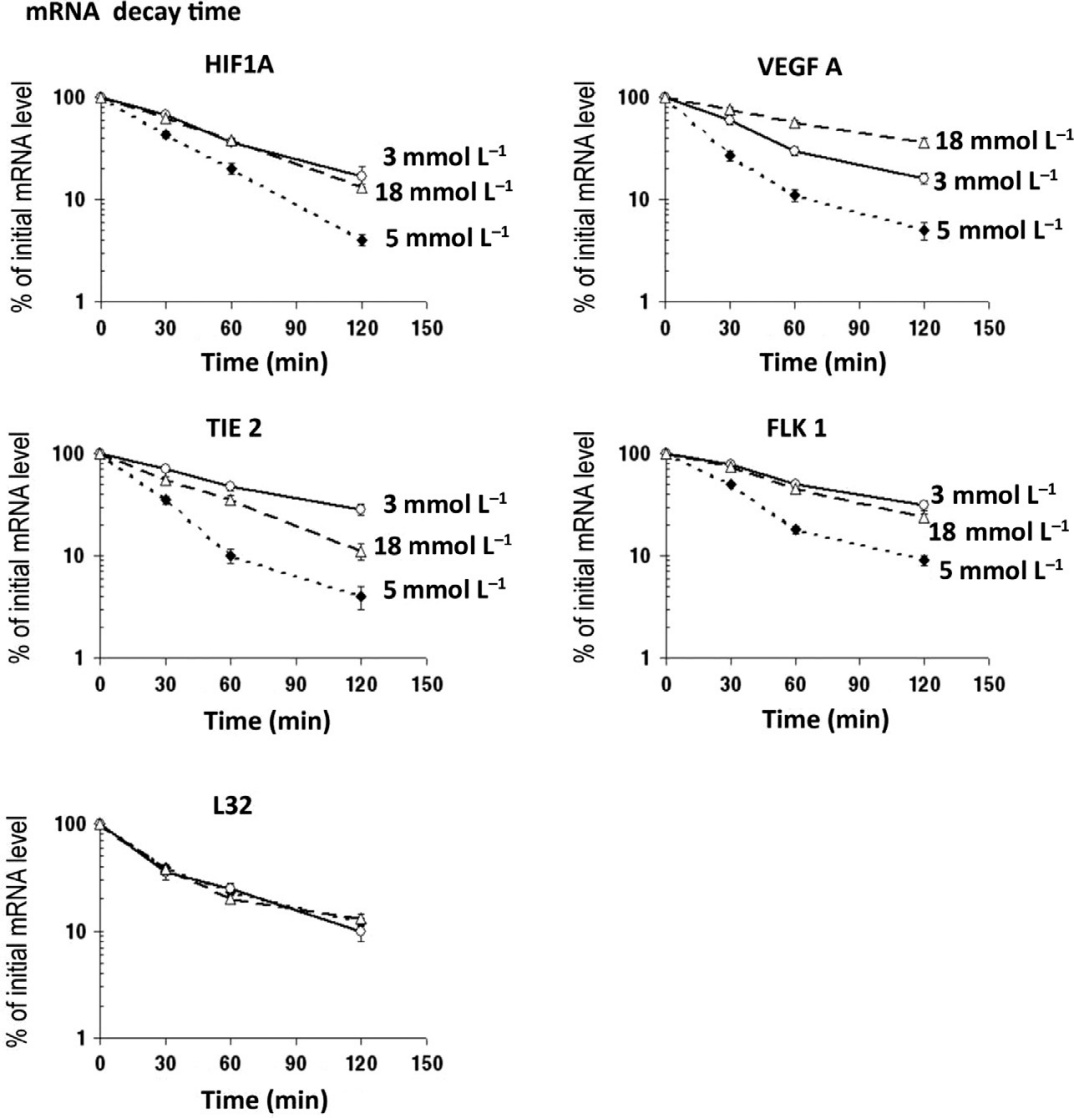
HIF1A, FLK1, TIE‐2 and VEGFA mRNAs are stabilized under low and high glucose. Overall transcription was inhibited by DRB in tubule suspensions under low (3 mmol/L, white circles), normal (5 mmol/L, black diamonds) or high (18 mmol/L, white triangles) glucose concentrations. The decay of mRNA levels was determined by RT‐real time PCR and normalized with 18S mRNA. As a control, the effect of glucose on L32 mRNA stability was determined in parallel. The results are plotted in log of the ratio to mRNA level at zero time, mean ± SD of four separate experiments, each point in duplicate.

Next, we asked whether increased stability accounts for the detected changes in transcripts levels upon glucose variations. To this purpose, overall transcription was inhibited with DRB. The resulting mRNA decays are shown in Fig. 6. HIF1A mRNA half‐life was approximately 25 min under normal glucose levels, but lengthened twofold after lowering glucose concentration to 3 mmol/L or after increasing it to 18 mmol/L. VEGFA transcripts, together with those of TIE‐2 and FLK1, were also affected by glucose variations. VEGFA mRNA half‐life lengthened from 12 min under normal glucose to 35 min under low glucose and to 60 min under high glucose. The same type of response was obtained with FLK1 and TIE‐2, but not with the mRNA encoding L32 ribosomal protein (Fig. 6). Therefore, these results show that glucose variations, and more strikingly low glucose microenvironment, stabilize HIF1A mRNA as well as VEGFA, TIE‐2, and FLK1 mRNAs.

## Discussion

We previously used isolated renal cortical tubules to show that oxygen deprivation induces changes in the level of a number of mRNAs (de Laplanche et al. 2006). Here, we used this model to investigate its adaptation to glucose shortage. The cortical tubular preparations are presumably fairly physiologic. However, as proximal tubules are not commonly thought to rely much, if at all, on glucose metabolism for energy production and transport virtually all glucose across the cells from apical to basolateral domains, one critical point is to verify that these preparations maintain a physiologic gluconeogenic, and nonglycolytic phenotype to be sure that we are not studying a dedifferentiated or pathologic condition. Although we did not monitor glucose transporters gene expression in the course of tubules incubation that would have been one way to control how physiological our preparations are, we checked under microscope the cohesion of the tubules incubated for up to 7 h under moderate hypoxia and different glycemia conditions. In these conditions, we did not see any dedifferentiation (data not shown). These results are consistent with the observation we previously made in the same cellular model (Braun et al. 2001). Hence, we had already validated the absence of dedifferentiation up to 6 h of moderate hypoxia despite glycemia variations, thereby leading us to postulate that hypoxia rather than glycemia may dedifferentiate the tubules.

Here, we first decided to analyze the level of mRNAs encoding proteins involved in angiogenesis (VEGFA, FLK1, TIE‐2) or glucose metabolism (GLUT1, Aldolase A). Our data show that high glucose and also low glucose environments increase levels of these mRNAs. Because these two responses occur in the same cell type, we hypothesize that this may reflect a novel window of regulation.

Here, we find out that VEGFA mRNA and a subset of mRNAs involved in angiogenesis and glucose consumption are upregulated under low glucose. We also notice that VEGFA and GLUT1 proteins, and to a somewhat similar tendency TIE‐2 protein, are upregulated under low glucose. Of note, in unstirred transformed renal tubular cells, total deprivation of glucose leads to VEGFA mRNA and protein increases, whereas HIF1A protein is absent (Bouvier et al. 2012). We further uncover that the upregulation of these mRNAs is likely HIFA‐independent. In support of these data, a subset of transcripts such as VEGFA has been reported to be translated under glucose deprivation, in spite of a general decrease in translation (Thomas and Johannes 2007) (Thomas et al. 2008).

Here, we show that HIF1A and HIF2A mRNAs levels respond to glucose whereas they do not correlate with oxygen supply. We further uncover that glucose variations, and more strikingly low glucose microenvironment, stabilize HIF1A mRNA as well as some of its transcriptional targets. Some links between HIFA mRNA levels and glucose shortage have been reported. HIF1A mRNA knockdown was associated with reduced glucose utilization after a period of 4 h hypoxia (Thomas et al. 2008). In a study of mRNAs to which ribosomes are bound under hypoxia, L32 was shown to belong to the mRNA subset that is less translated, whereas HIF1A mRNA continued to be translated (Thomas and Johannes 2007).

The poly(A)‐binding protein interacting protein 2 (PAIP2) is sometimes considered as a downregulating protein as it decreases cap‐dependent, non‐Internal Ribosome Entry Site (IRES)‐dependent translation that is regulated by mTOR (McLaughlin and Hime 2011). However, PAIP2 is also able to bind some IRES‐containing transcripts such as those of virally induced proteins (Soto Rifo et al. 2007). IRES have been identified in TIE‐2 (Park et al. 2006), HIF1A (Lang et al. 2002), and VEGFA mRNAs (Akiri et al. 1998), and several lines of evidence showed that VEGFA and HIF1A IRES are used to maintain their translation under ischemic conditions (Schepens et al. 2005; Bornes et al. 2007). Because we here found VEGFA and HIF1A mRNAs to be stabilized under glucose shortage, and because VEGFA mRNA was reported to be stabilized through its association with PAIP2 (Onesto et al. 2004), it would be interesting to further investigate whether VEGFA and HIF1A mRNAs could bind PAIP2 to be stabilized.

In summary, this work demonstrates for the first time that in normal, nontransformed renal tubule, 1) glucose shortage increases VEGFA mRNA and protein levels in spite of high oxygen supply, 2) glucose shortage upregulates a subset of messengers involved in angiogenesis, including HIF1A, TIE‐2, and FLK1 mRNAs, 3) this subset is regulated by mRNA stabilization independently of HIF proteins, 4) the proteins produced by VEGFA and GLUT1 mRNAs are also similarly sensitive to low glucose.

## Acknowledgments

Thanks are due to Catherine Godinot, Eric Clottes, Marc Billaud, Jean‐Yves Scoazec, Gilles Pagès, and David Bernard for helpful discussions. We gratefully acknowledge Abdallah Gharib for his participation in animal experiments, Jocelyne Demont for skillful technical assistance and David Cox for careful reading of the manuscript.

## Conflict of Interest

None declared.
